# A Port‐A‐Cath in aorta

**DOI:** 10.1002/ccr3.1496

**Published:** 2018-03-22

**Authors:** Dominika Zoltowska, Jagadeesh Kalavakunta

**Affiliations:** ^1^ Department of Internal Medicine Western Michigan University Homer Stryker School of Medicine Kalamazoo Michigan; ^2^ Department of Cardiology Michigan State University/Borgess Medical Center Kalamazoo Michigan

**Keywords:** Arterial injury, interventional radiology, malpositioned central venous catheter, Port‐A‐Cath

## Abstract

Totally implantable venous access ports are valuable invention for oncological patients. Erroneous arterial malposition rate is estimated from 1.1% to 3.7% (Bowen et al. *Am. J. Surg.,* 2014, 208, 937). Early recognition and management are crucial to prevent further complications.

## Case

**Figure 1:**
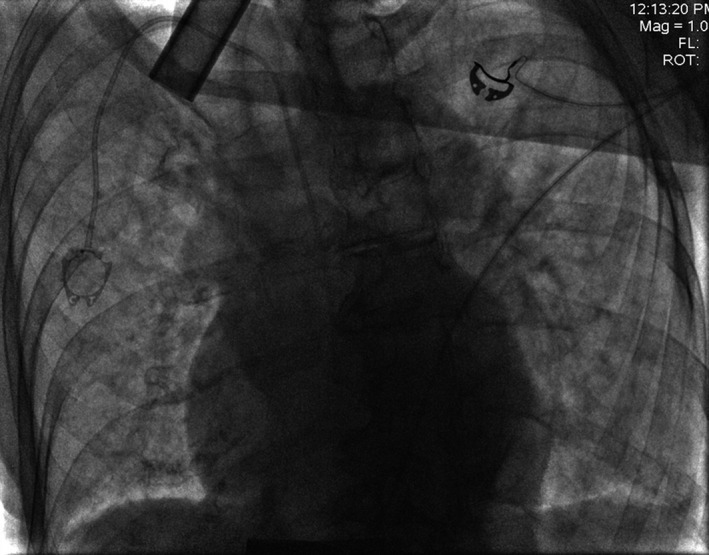
Chest radiography after initial right Port‐A‐Cath placement.

**Figure 2:**
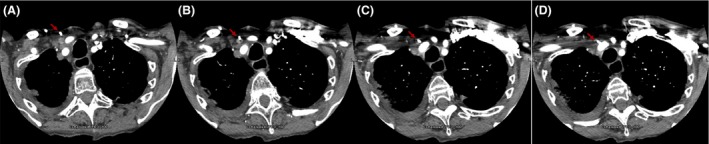
CT angiogram transverse views A‐D. Red arrow indicating the Port‐A‐Cath entering the right subclavian artery.

**Figure 3:**
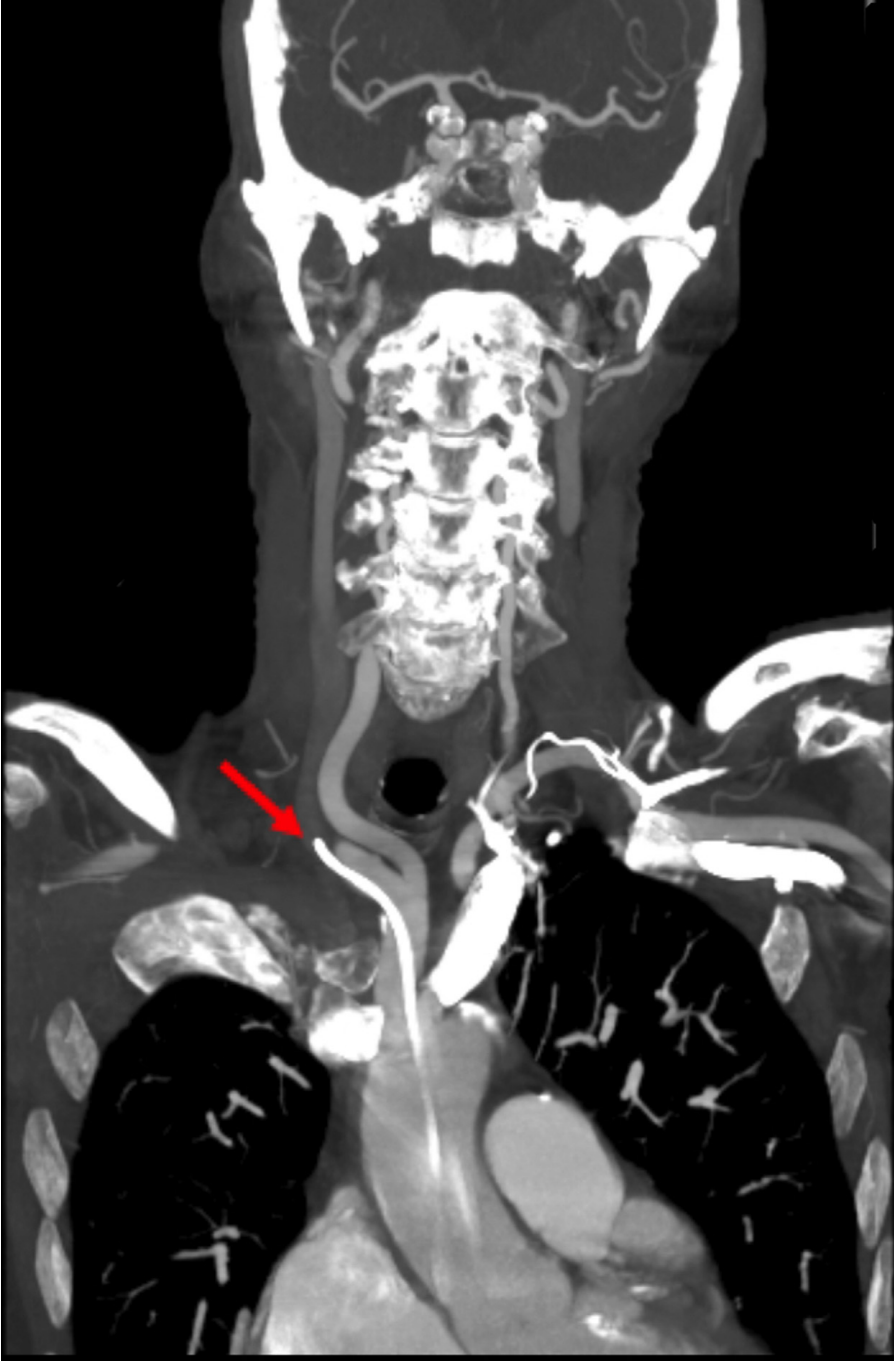
CT angiogram (coronal view). Red arrows showing the malposition Port‐A‐Cath.

**Figure 4:**
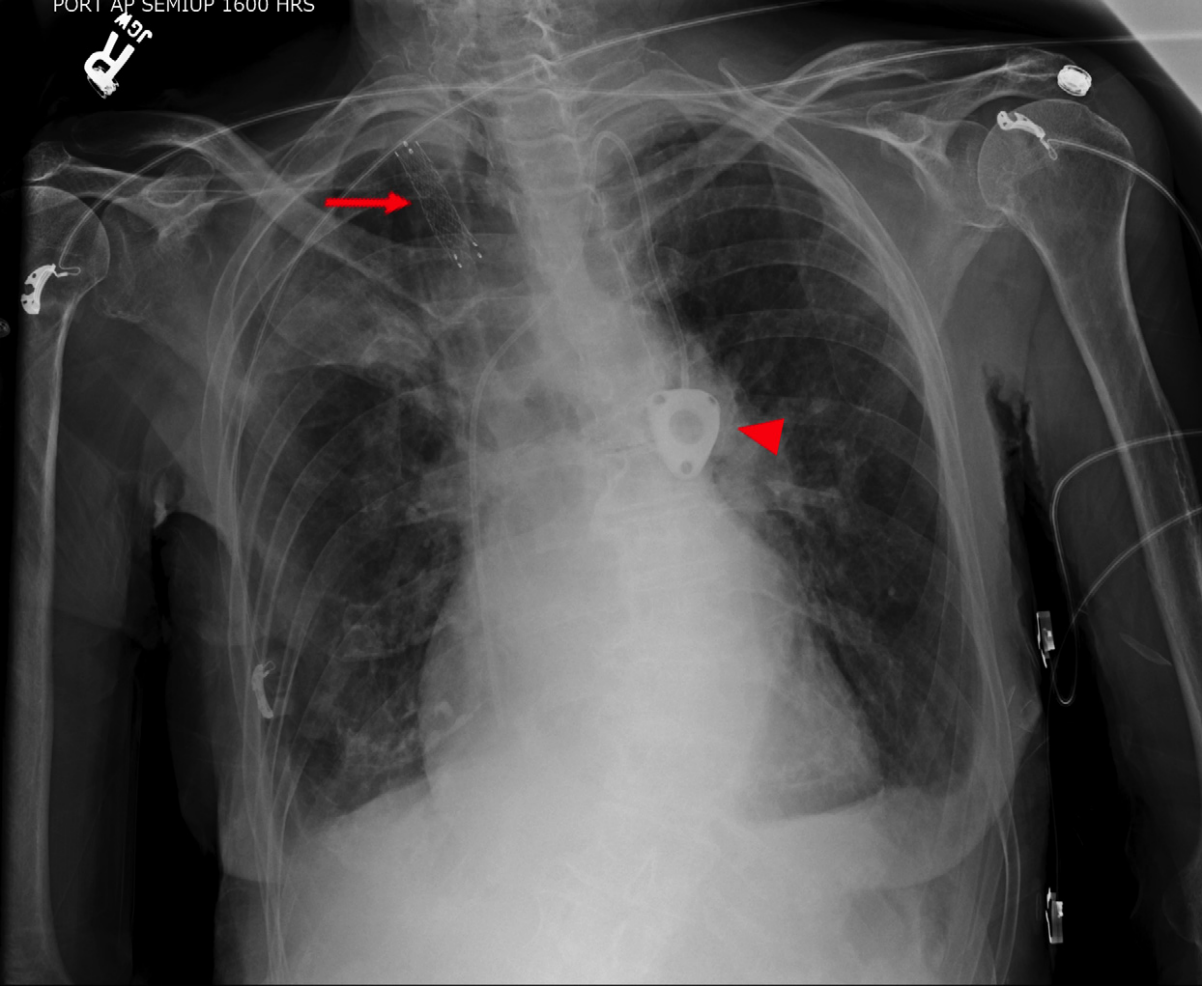
Chest radiogram obtained after the subsequent procedure. A new Port‐A‐Cath (arrowhead) and the stent in RSA are visualized (arrow).

A 89‐year‐old female with metastatic Merkel cell carcinoma underwent a central venous port placement (Port‐A‐Cath) for chemotherapy 1. Procedure was performed by right internal jugular vein (RIJ) approach under ultrasound and fluoroscopic guidance. A satisfactory position was documented (Fig. 1). The port was tested and found to flush and aspirate appropriately. The next day, patient complained of a new onset sharp midsternal chest pain and shortness of breath. A computed tomography (CT) angiogram revealed a right‐sided Port‐A‐Cath perforating RIJ and entering the right subclavian artery (RSA) (Fig. 2) with a tip localized in the aorta (Fig. 3). No contrast extravasation was noted. Subsequently, patient underwent endovascular retrieval of the malposition Port‐A‐Catheter combined with a stent graft placement into the RSA without complications (Fig. 4).

## Clinical Question

What are potential complications of erroneous arterial placement of central venous catheter?

Erroneous arterial cannulation may lead to hematoma, pseudoaneurysm, arteriovenous fistula, dissection, hemorrhage, extremity ischemia, embolization, and even death 2.

## Conflict of Interest

None declared.

## Authorship

DZ and JK: were the physicians in charge of the patient throughout hospitalization and follow‐up. DZ: prepared the manuscript draft, which was critically revised and approved by JK.
